# Comment on “A Case of Healthcare Associated Pneumonia Caused by *Chryseobacterium indologenes* in an Immunocompetent Patient”

**DOI:** 10.1155/2015/730548

**Published:** 2015-09-07

**Authors:** Panagiotis Papastergiou

**Affiliations:** Path Links Pathology Service, Microbiology Department, Northern Lincolnshire and Goole NHS Foundation Trust, Lincoln County Hospital, St. Anne's Road, Lincoln, Lincolnshire LN2 5RF, UK

I am writing regarding the publication of* S. A. Nemil et al.*, “A Case of Healthcare Associated Pneumonia Caused by* Chryseobacterium indologenes* in an Immunocompetent Patient” [1]. In the published case report, the authors state that the patient acquired a Healthcare Associated Pneumonia (HAP) caused by* Chryseobacterium indologenes*. Notwithstanding that the patient had a HAP, the evidence that the infection was caused by* C. indologenes* is insufficient and could possibly be questionable.

(a) According to the authors both* E. coli *and* C. indologenes* were isolated from a transtracheal aspirate (TTA) sample; however it is suggested that the infection was caused only by* C. indologenes*.

(b) The transtracheal aspirate (TTA) samples are ideal for bacteriology investigation; however an organism growth on a single TTA sample is not proof of the pathogen that causes the infection. The interpretation of the relevance of bacterial growth depends on the pathogen and it is high if the isolate is a typical pathogenic organism that causes respiratory tract infection/chest infection and significantly low if the isolated organism is an uncommon bacterium (*Chryseobacterium indologenes*).* Chryseobacterium indologenes* is an opportunistic pathogen, not normally present in the human microflora, although it is widely distributed in nature. The patient was not immunocompromised and therefore is more susceptible to an organism with low pathogenicity. Important information regarding the microbiology culture growth (quantitative or semiquantitative) and presence of inflammation were not reported in the document in order to estimate the significance of the culture. The presence of two organisms on the same culture reduces further the clinical significance of the culture result. In addition the diagnostic utility of TTA is likely decreased in a patient with a chronic airway disease, such as bronchiectasis, in which case a false-positive culture result should be expected due to common colonization below the level of the larynx [2].

(c) According to the authors the antimicrobial susceptibility test revealed that the “*C. indologenes was resistant to carbapenems and sensitive to levofloxacin. Levofloxacin was added to antimicrobial therapy on the 6th day of admission. On follow-up, after the second day of therapy modification, improvement on blood gas parameters was observed and the patient was extubated.*” The authors speculate that the patient had an infection caused by* C. indologenes* due to the patient not responding to the previous antibiotic treatment. The patient's antimicrobial therapy was modified to ertapenem on the 3rd day of admission (when urine culture yielded ESBL and after 3 days treatment with piperacillin-tazobactam), possibly indicating that the patient was possibly close to or less than 72 hours on ertapenem treatment when levofloxacin was added. It would be interesting to know if the 82-year-old patient had hypoalbuminaemia. Hypoalbuminaemia is rarely considered in clinical decisions but ertapenem is highly albumin bound (85–95%) and data from multiple studies showed that hypoalbuminaemia may have a profound effect on pharmacokinetics (failure to attain pharmacodynamic targets) and therefore could possibly lead to treatment failure or a delayed clinical response [3, 4]. In addition a large proportion of* C. indologenes* isolates are susceptible to piperacillin-tazobactam (the patient was initially treated with piperacillin-tazobactam) [5, 6]; however the* C. indologenes* susceptibility to piperacillin-tazobactam is not documented.

On conclusion, the interpretation of the relevance of bacterial growth of uncommon bacterium with low pathogenicity (*C. indologenes*) in transtracheal aspirates needs to be carefully interpreted, including clinical cases when there is a clear clinical improvement after targeted antimicrobial therapy to the uncommon bacterium has been applied.

## References

[1] Nemli S. A., Demirdal T., Ural S. (2015). A case of healthcare associated pneumonia caused by *Chryseobacterium indologenes* in an immunocompetent patient. *Case Reports in Infectious Diseases*.

[2] Bartlett J. G. (2011). Diagnostic tests for agents of community-acquired pneumonia. *Clinical Infectious Diseases*.

[3] Roberts J. A., Pea F., Lipman J. (2013). The clinical relevance of plasma protein binding changes. *Clinical Pharmacokinetics*.

[4] Ulldemolins M., Roberts J. A., Rello J., Paterson D. L., Lipman J. (2011). The effects of hypoalbuminaemia on optimizing antibacterial dosing in critically ill patients. *Clinical Pharmacokinetics*.

[5] Kirby J. T., Sader H. S., Walsh T. R., Jones R. N. (2004). Antimicrobial susceptibility and epidemiology of a worldwide collection of *Chryseobacterium* spp.: report from the SENTRY antimicrobial surveillance program (1997–2001). *Journal of Clinical Microbiology*.

[6] Chen F.-L., Wang G.-C., Teng S.-O., Ou T.-Y., Yu F.-L., Lee W.-S. (2013). Clinical and epidemiological features of *Chryseobacterium indologenes*infections: analysis of 215 cases. *Journal of Microbiology, Immunology and Infection*.

